# Early intermittent low-dose sclerostin antibody treatment promotes surface bone formation and reduces bone loss, but also decreases osteocyte apoptosis and mechanotransduction in ovariectomized rats: A pilot study

**DOI:** 10.1016/j.bonr.2026.101915

**Published:** 2026-04-07

**Authors:** Syeda Masooma Naqvi, Wahaaj Ali, Hollie Allison, Laura M. O'Sullivan, Gill Holdsworth, Juan Alberto Panadero-Perez, Jessica Schiavi-Tritz, Laoise M. McNamara

**Affiliations:** a Mechanobiology and Medical Devices Research Group (MMDRG), Biomedical Engineering, University of Galway, Ireland; b Laboratory in Reactions and Chemical Engineering, CNRS-Lorraine University, UMR 7274, Vandœuvre-lès-Nancy, France; c Early Solutions, UCB Pharma, Slough, UK

**Keywords:** Sclerostin antibody (Scl-Ab), Postmenopausal osteoporosis, Ovariectomized rats, Bone remodelling, Secondary mineralization, Early intervention

## Abstract

Neutralizing sclerostin antibodies (Scl-Ab) mitigate bone loss and promote bone formation to address fracture risk in postmenopausal osteoporosis. Clinically, this treatment is administered monthly for women at high risk of fragility fractures, who are often years into menopause. Preclinical studies have demonstrated that dampening of bone formation occurs with continuous dosing at supraphysiological doses. Osteoporotic bone loss occurs rapidly during early menopause, followed by longer-term changes in bone mineralization and osteocyte activity. Whether earlier administration of lower-exposure Scl-Ab can mitigate bone loss and osteocyte-mediated mineralization is unknown. The objective of this study was to evaluate the effects of early intermittent low-dose Scl-Ab on: (1) osteoclastogenesis and bone resorption, (2) perilacunar remodelling, (3) secondary mineralization, and (4) osteocyte mechanosensitivity. Female retired breeder Wistar rats underwent bilateral ovariectomy and received monthly low-dose Scl-Ab injections (2 mg/kg/month) from 3 to 14 weeks post-OVX, while a control group remained untreated. Early intermittent low-dose Scl-Ab treatment increased bone formation and reduced osteoclastogenesis and catabolic gene expression ((Sost, Ctsk, Mmp9) compared to untreated rats. Treatment also decreased the percentage of empty lacunae and the number of MMP14+ osteocytes, accompanied by lower perilacunar mineral density and smaller lacunar size, indicating improved osteocyte survival and reduced perilacunar remodelling. Conversely, expression of osteocyte-mediated mineralization genes (DMP1, PHEX, OPN, ALP) and mechanotransduction-related genes (Vcl, integrins α5, αV, β1, CX43, Axin2, IFT88, Adcy6, Pkd1, Cav1) were reduced. Together, these findings suggest that early intermittent low-dose Scl-Ab therapy promotes surface bone formation while attenuating osteocyte-mediated mineralization after initial bone loss.

## Highlights

• Early low-dose sclerostin antibody reduces osteocyte apoptosis after ovariectomy
• Intermittent Scl-Ab suppresses perilacunar remodelling and secondary mineralization
• Osteoclastogenesis is attenuated despite conservative Scl-Ab dosing
• Osteocyte-associated mechanotransduction gene expression is altered early
• Bone mass changes are modest, consistent with an exploratory low-dose regimen

## 1 Introduction

Postmenopausal osteoporosis is characterized by excessive bone resorption, followed by changes in bone tissue mineralization and osteocyte function (O'Sullivan et al., 2020; Emerton et al., 2010; Allison et al., 2022). Rapid trabecular bone loss occurs during the first two years following menopause, slowing thereafter to approximately 1% per year (Sowers et al., 2010). This early phase is associated with accelerated bone turnover and structural deterioration (Gregson et al., 2022; Saag et al., 2017). In rodent models, estrogen deficiency induces early trabecular bone loss within four weeks post-ovariectomy, followed by prolonged secondary mineralization characterized by increased matrix mineral density, microporosity, and perilacunar remodelling (O'Sullivan et al., 2020; Emerton et al., 2010; Allison et al., 2022). This secondary mineralization may be driven by maturation and accumulation of mineral within the bone matrix. Osteocytes can also actively regulate local mineral composition through perilacunar remodelling and micropetrosis. It has been reported that osteocyte apoptosis and perilacunar resorption contribute to localized hypermineralization (micropetrosis) and reduced bone toughness (Emerton et al., 2010; Allison et al., 2022; Kogawa et al., 2018). However, strategies to inhibit pathological mineralization mediated by osteocytes have not yet been explored.

Osteocytes synthesize and release sclerostin, a glycoprotein that plays a central role in regulating osteoclast resorption and osteoblast matrix and mineral deposition. Sclerostin inhibits canonical Wnt/β-catenin signalling, suppressing osteoblast differentiation and bone formation (Notsu et al., 2017; Bonewald, 2011), while indirectly promoting bone resorption through osteoblast–osteoclast coupling (Ominsky et al., 2017a; Taylor et al., 2016). Sclerostin expression increases during mechanical unloading, suppressing bone formation and thereby contributing to reduced bone strength (Kogawa et al., 2018; Kogawa et al., 2013). These features make sclerostin a key therapeutic target in postmenopausal osteoporosis. Neutralizing antibodies against sclerostin (Scl-Ab) stimulate bone formation (Padhi et al., 2014) by promoting osteogenic differentiation of progenitor cells and activating bone-lining cells (Padhi et al., 2014; Cosman et al., 2016; McClung et al., 2014; Costa et al., 2021), while indirectly suppressing osteoclast activity (Ominsky et al., 2017a; Taylor et al., 2016). Sclerostin antibody treatment can influence bone mineralization kinetics, including the maturation of mineral within newly formed bone matrix (Saag et al., 2017; Cosman et al., 2016; McClung et al., 2014; Ross et al., 2014). However, its effects on osteocyte-mediated mineralization and mechanosensory function during early estrogen deficiency remains poorly understood. Clinical studies in postmenopausal women show that monthly romosozumab administration (210 mg) significantly increases bone mineral density (BMD) and reduces vertebral and non-vertebral fracture risk within the first year (Saag et al., 2017; Cosman et al., 2016; McClung et al., 2014). Single doses rapidly increase serum P1NP and decrease sCTX, indicating enhanced formation and reduced resorption (Padhi et al., 2011). However, continued monthly administration beyond six months leads to a partial return of bone formation markers (P1NP, osteocalcin, ALP) toward baseline, suggesting dampened anabolic responses with prolonged exposure (Taylor et al., 2016; Holdsworth et al., 2018; McClung et al., 2018). Treatment-free periods restore bone formation (Taylor et al., 2016; Holdsworth et al., 2018; McClung et al., 2018), indicating that intermittent dosing, with breaks in antibody administration may preserve efficacy, though the underlying mechanisms are not fully understood.

Mechanistic insights come from preclinical studies in rodents and nonhuman primates. Weekly or twice-weekly Scl-Ab administration in ovariectomized (OVX) rats and cynomolgus monkeys, restores trabecular and cortical bone mass, enhances periosteal and endocortical formation, and improves mechanical strength (Ominsky et al., 2017a; Virdi et al., 2015; Wu et al., 2018; Stolina et al., 2014; Li et al., 2014; Ominsky et al., 2017b; Turk et al., 2020). These studies use higher doses (≥25 mg/kg in rodents) than the clinical equivalent (∼3 mg/kg weekly), due to faster IgG clearance (Taylor et al., 2016; Ominsky et al., 2017b; Turk et al., 2020). Prolonged treatment normalizes Wnt target genes and increases secreted antagonists, such as SOST, consistent with feedback regulation observed in humans (Taylor et al., 2016; Holdsworth et al., 2018). Despite these advances, the efficacy of low-dose or intermittent Scl-Ab regimens remains incompletely understood, motivating the exploration of whether lower-exposure schedules can preserve anabolic responses while minimizing feedback dampening.

At the cellular level, osteocytes are key mediators of these effects. Estrogen deficiency reduces osteocyte mechanosensitivity characterized by diminished intracellular calcium oscillations and attenuated responses to mechanical loading (Deepak et al., 2017). These changes exacerbate osteocyte-mediated osteoclastogenesis and bone resorption (Geoghegan et al., 2019; Allison and McNamara, 2019; Simfia et al., 2020). Sclerostin inhibition reverses these effects, restoring osteoblast and osteocyte activity via the Wnt/β-catenin signalling pathway. In OVX rats, Scl-Ab treatment upregulates Wnt target and ECM-related genes, while in mice, short-term Scl-Ab exposure increases ECM-associated transcripts such as *Twist1* and *Cgref* (Kedlaya et al., 2013). Furthermore, long-term estrogen deficiency alters extracellular matrix gene expression, bone tissue mineral distribution, and the lacunar-canalicular network indicative of perilacunar remodelling and disrupted mechanosensory capacity (Naqvi et al., 2024). Human studies corroborate these findings, showing more heterogeneous bone mineral distribution and increased hypermineralized regions under estrogen deficiency (Parle et al., 2020). However, the effectiveness of Scl-Ab treatment during early bone loss, before secondary mineralization occurs, remains unexplored. We hypothesize that early Scl-Ab treatment may prevent secondary mineralization by targeting perilacunar resorption and osteocyte apoptosis in postmenopausal osteoporosis.

The objective of this study was to determine whether initiating monthly low-dose Scl-Ab treatment three weeks post-ovariectomy could: (1) mitigate osteoclastogenesis and bone resorption, (2) preserve perilacunar remodelling, (3) prevent secondary mineralization, and (4) maintain osteocyte mechanosensitivity during estrogen deficiency.

## 2 Methods

### 2.1 Animal model

Female retired breeder Wistar rats (6 months old, Charles River, Ireland) underwent bilateral ovariectomy (*n* = 8), confirmed by necropsy. To study subcutaneous delivery of intermittent low-dose Scl-Ab (Turk et al., 2020) (rodent version of romosozumab: Scl-Ab; r13c7, UCB Pharma, UK / Amgen Inc., UK), ovariectomized rats were assigned to either (a) no treatment (Control, *n* = 4, body weight: 382 ± 39 g) or (b) Scl-Ab (2 mg/kg/month, n = 4, body weight: 399 ± 49 g), administered every 4 weeks starting 3 weeks post-ovariectomy. This 3-week time point corresponds to the initial phase of rapid bone loss but precedes secondary mineralization changes (O'Sullivan et al., 2020). The 2 mg/kg monthly Scl-Ab regimen was intentionally selected as a low-exposure exploratory dose, lower than the approximate clinical equivalent of 3 mg/kg monthly (210 mg romosozumab in humans). Most preclinical studies use 25–50 mg/kg weekly (Taylor et al., 2016; Ominsky et al., 2017b; Turk et al., 2020), representing supraphysiologic exposures relative to humans. Our goal was to evaluate osteocyte-specific responses under a conservative, early-intervention dosing schedule, rather than to reproduce maximal pharmacological effects. Scl-Ab was prepared in sterile phosphate-buffered saline (PBS) for subcutaneous injection. Euthanasia occurred at week 14 post-OVX (three weeks after the final Scl-Ab injection), corresponding to 11 weeks of treatment exposure. All outcome measures were assessed at this single terminal timepoint. Procedures followed guidelines from the University of Galway's Animal Care and Research Ethics Committee (ACREC) and the Health Products Regulatory Authority (HPRA).

### 2.2 Calcein labelling

To label actively mineralizing bone surfaces animals received two intraperitoneal calcein injections (20 mg/kg) at 14 and 4 days before euthanasia. This enabled quantification of mineral apposition rate (MAR) over a 10-day interval. At week 14, vertebrae were isolated from Control (*n* = 4) and Scl-Ab (n = 4) rats, fixed in 4% paraformaldehyde, and processed for analysis. Trabecular bone within the lumbar vertebral body (L4) was sectioned using a diamond-blade low-speed saw (Isomet™, Buehler, IL, USA), embedded in epoxy, cut into 1.5-mm slices, and mounted on SuperFrost® Plus slides (Menzel Gläser). Calcein-labelled sections were imaged by fluorescence microscopy at ×20 magnification and calibrated (μm/pixel). The total bone surface length (BS) within the trabecular region of interest (ROI) was traced, and the lengths of double-labelled (dL) and single-labelled (sL) surfaces were measured along the same BS using ImageJ. Mineralizing surface per bone surface (MS/BS) was calculated as MS/BS = [(dL + 0.5 × sL) / BS] × 100. Mineral apposition rate (MAR, μm/day) was determined from double labels as the mean inter-label distance divided by the 10-day interval between injections. Bone formation rate per bone surface (BFR/BS) was then calculated as BFR/BS = MAR × (MS/BS fraction). For each animal, measurements were averaged across three sections and at least five fields per section; group means ± SD are reported. All analyses were performed blinded to treatment.

### 2.3 In vivo and ex vivo nano-CT imaging and analysis

In vivo micro-CT scanning (VivaCT40, Scanco Medical AG) was conducted to investigate the effect of Scl-Ab on morphometry and mineralization during estrogen deficiency. Scans were performed at day 0, week 4, week 8 and week 14 on the right tibia of Control (*n* = 4) and Scl-Ab (n = 4) groups at 15 μm resolution. Scans were not performed at week 3 (Scl-Ab treatment onset), baseline bone status at this time point was therefore assumed based on previously published OVX data from our laboratory (O'Sullivan et al., 2020). The X-ray settings, region of interest, and segmentation threshold were applied according to a previously established protocol (O'Sullivan et al., 2020). Analyses were conducted on the proximal tibia (trabecular VOI). Microarchitectural parameters (Bone volume fraction (BV/TV), trabecular number (Tb.N), trabecular thickness (Tb.Th), and trabecular spacing (Tb.Sp)) were quantified from the 3D reconstruction of the trabecular volume of interest using evaluation scripts in the Scanco Image Processing Language in the proximal tibia of the treatment groups at each time point.

Ex vivo high-resolution nano-CT scanning (Zeiss Xradia Versa 620) was performed at week 14 on right tibiae from control (*n* = 4) and Scl-Ab (n = 4) groups. Hydroxyapatite (HA) phantoms (QRM, PTW Dosimetry) were used to calibrate intensity measurements. Scans were conducted using a 0.4× objective at 70 kV, 8.5 W, with an LE3 source filter at an isotropic resolution of 9.14 μm. A 3-mm region below the proximal tibial growth plate was analysed to assess trabecular bone microarchitecture; BV/TV, Tb.N, Tb.Th, and Tb.Sp.

Lacunae and peri-lacunar mineral density were also assessed using ultra-high-resolution scans (1.11 μm voxel size). Trabecular bone sections (0.5 mm thick) were prepared, mounted, and scanned with a 4× objective and a 4-s exposure time with same electrical parameters. Lacunae were segmented using Otsu thresholding quantification of peri-lacunar mineral density and lacunar surface area.

### 2.4 Histological and immunohistochemical analysis

Histological assessments (TRAP, H&E, and MMP14 immunohistochemistry) were performed on distal femoral metaphyseal sections. At week 14, femurs from Control and Scl-Ab rats were fixed in 4% paraformaldehyde. Distal femoral metaphyseal sections (1.5 mm) were cut using a diamond blade saw and decalcified in 10% EDTA for 14 days. Decalcification was confirmed via oxalate and physical probing tests. Samples were rinsed in PBS overnight, processed using a Leica ASP300 tissue processor and embedded in paraffin (Leica EG1150H). Paraffin sections (8 μm) were cut using a Leica RM2235 microtome, mounted on SuperFrost® Plus slides, and stored at room temperature, until staining.

TRAP and H&E staining were performed to visualize osteoclast number and osteocytes within lacunae, respectively. Images were captured using a light microscope (Olympus BX43, Olympus, Japan), and quantitative analysis was conducted on ImageJ.

For immunohistochemistry, paraffin-embedded sections were incubated overnight at 4 °C with primary anti-MMP14 antibody (1:100; Abcam, ab38971), followed by a secondary antibody (Goat Anti-Rabbit IgG H&L (HRP); Abcam, ab6721) for 1 h at room temperature. Slides were developed using DAB substrate chromogen solution for 5 min. Control sections, received antibody diluent instead of the primary antibody, were processed alongside experimental samples to confirm specificity. The prevalence of MMP14+ osteocytes, normalized to total bone area, was assessed using ImageJ (Dole et al., 2017).

### 2.5 Gene expression analysis

Gene expression analysis was carried out on cortical bone isolated from the tibial diaphysis. Tibiae from control (*n* = 4) and Scl-Ab (n = 4) groups were dissected, washed with DNase/RNase-free PBS, and cortical bone isolated. Samples were washed to remove bone marrow, snap-frozen in liquid nitrogen, and stored at −80 °C. Total RNA was extracted using the RNeasy Fibrous Tissue Mini Kit (Qiagen) and converted to cDNA with the SuperScript VILO Kit (Invitrogen). RNA quality was assessed by 260/280 and 260/230 absorbance ratios. Scl-Ab-treated samples showed acceptable 260/280 ratios (1.8–2.0), with slightly lower 260/230 ratios (<2), but RNA quality was sufficient for mRNA microarray analysis, confirmed by consistent signal intensities and reproducible biological replicates.

TaqMan® microarray analysis was performed using Applied Biosystems TaqMan® Array (format: FAST 96-well) on a StepOnePlus™ PCR system, with Rpl13a as the reference gene. The study examined gene expression related to bone resorption and matrix degradation (RANKL, OPG, Sost, NFATc-1, CtsK, MMP9, MMP13, Ccr2, ATG7, Raptor), apoptosis (Tp53, Casp3, Lamp1), bone formation and matrix production (OPN, Col1α1, Col1α2, OCN, Alp (osteoblast-associated) and DMP1, PHEX, TGFβ1, Klf10 (osteocyte-enriched)), other regulators (Ager, Fn1 (stress- and ECM-associated)) and mechanosensation and mechanotransduction (VCL, TRPV4, ADCY6, AXIN2, ITGβ1, ITGα5, ITGαV, CX43, RYR, PKD1, CAV1). Results are expressed as relative quantitative changes normalized to age-matched OVX controls (Control), with normalization to the housekeeping gene Rpl13a. Eight microarrays were performed, equally divided between Control and Scl-Ab groups.

### 2.6 Statistical analysis

Statistical analyses were performed using GraphPad Prism. Differences between Control and Scl-Ab groups were assessed via Student's *t*-tests, with significance set at *p* ≤ 0.05. CT parameters (BV/TV, Tb.N, Tb.Th, Tb.Sp) were tested for normality; normally distributed data were analysed with t-tests, while non-normal data were analysed using Mann-Whitney tests. Two-way ANOVA was conducted to assess time and treatment interactions, followed by Tukey's post hoc tests for significant interactions. Results are presented as mean ± SD. This study was designed as a pilot exploratory experiment with small group sizes (*n* = 4).

## 3 Results

### 3.1 Bone formation recovery in Scl-Ab treatment of ovariectomy-induced bone loss

**Fig. 1:**
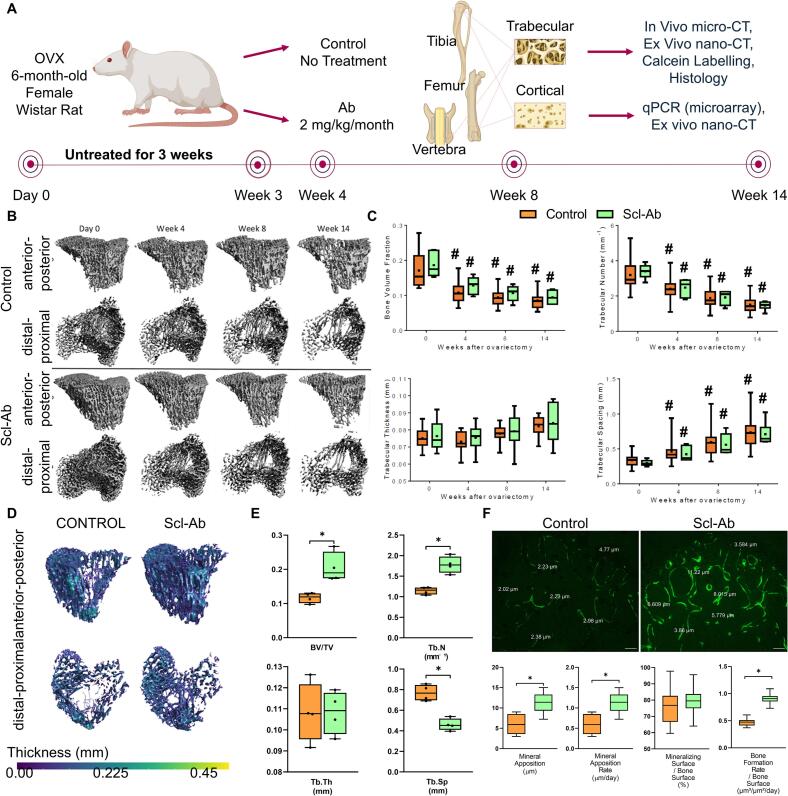
Surface Bone formation in early low-dose Scl-Ab treatment of ovariectomy-induced bone loss. (A) Study design: Female retired breeder Wistar rats were ovariectomized at 6 months. Three weeks post-ovariectomy, the animals were randomly assigned to (i) Control (no treatment) or (ii) Scl-Ab (systemic delivery of 2 mg/kg/month Scl-Ab). (B) 3D micro-CT images of proximal tibial trabecular bone at 0, 4, 8, and 14 weeks post-OVX in both groups, shown in two orthogonal views: anterior-posterior and distal-proximal. (C) Comparison of bone mass and microarchitectural parameters in Control and Scl-Ab groups at 0, 4, 8, and 14 weeks post-OVX. (D) High-resolution 3D nano-CT images of metaphyseal trabecular bone from the proximal tibia of a representative Control and Scl-Ab group at 14 weeks post-OVX, shown in two orthogonal views: anterior-posterior and distal-proximal. (E) Comparison of bone mass and microarchitectural parameters between the Control and Scl-Ab groups 14 weeks post-OVX. (F) Calcein labelling of surface bone formation in Control and Scl-Ab groups, mineral apposition (◻m) and rate (μm/day) quantified (*n* = 4/group). Box plots show median, IQR, and range. *p < 0.05; #p < 0.05 vs. baseline (day 0). Scale = 50 μm. Data in panels B–E from trabecular bone of the proximal tibia; calcein labelling (F) in trabecular bone of a lumbar vertebra (L4).

To assess the longitudinal effects of Scl-Ab treatment on bone microarchitecture and mineralization, we performed both in vivo micro-CT and ex vivo nano-CT analyses (Fig. 1A). Morphometric analysis by in vivo micro-CT (15 μm) indicated no significant differences in bone volume fraction and trabecular microarchitecture between Scl-Ab and Control groups at baseline (day 0), week 4, week 8 and week 14 (Fig. 1B, C). However, there was a significant decrease in bone volume fraction and trabecular number and a significant increase in trabecular spacing in week 14 compared to day 0 for both Control and Scl-Ab groups.

Morphometric analysis at nano-CT resolution (9.14 μm) revealed significant differences between the Scl-Ab treated and Control groups at week 14 (Fig. 1D, E). Specifically, there was a significant increase in BV/TV and trabecular number, and a significant decrease in trabecular spacing in the Scl-Ab group compared to the untreated OVX group. No significant differences were observed in trabecular thickness.

Fluorochrome labelling of mineral apposition (Fig. 1F) confirmed that Scl-Ab therapy enhanced bone formation in OVX rats. The mineral apposition was 11.54 ± 2.194 μm in the Scl-Ab-treated group compared to 6.16 ± 2.347 μm in untreated OVX controls (*p* < 0.05). The mineral apposition rate was 1.14 ± 0.22 μm in the Scl-Ab-treated group compared to 0.62 ± 0.23 μm in untreated OVX controls (p < 0.05).

Dynamic histomorphometry revealed that Scl-Ab treatment increased the extent of actively mineralizing surfaces and the overall bone formation rate. Mineralizing surface per bone surface (MS/BS) was higher in Scl-Ab-treated rats (79.6 ± 7.3%) compared with OVX controls (75.5 ± 10.1%), although this was not significant. Bone formation rate per bone surface (BFR/BS) was almost doubled in the Scl-Ab group (0.91 ± 0.08 μm^3^/μm^2^/day) relative to controls (0.47 ± 0.06 μm^3^/μm^2^/day; *p* < 0.05). These data confirm that early intermittent Scl-Ab treatment enhances both the rate and extent of trabecular bone formation.

### 3.2 Osteoclastogenesis and bone resorption are reduced in OVX rats treated early with intermittent low-dose Scl-Ab

**Fig. 2:**
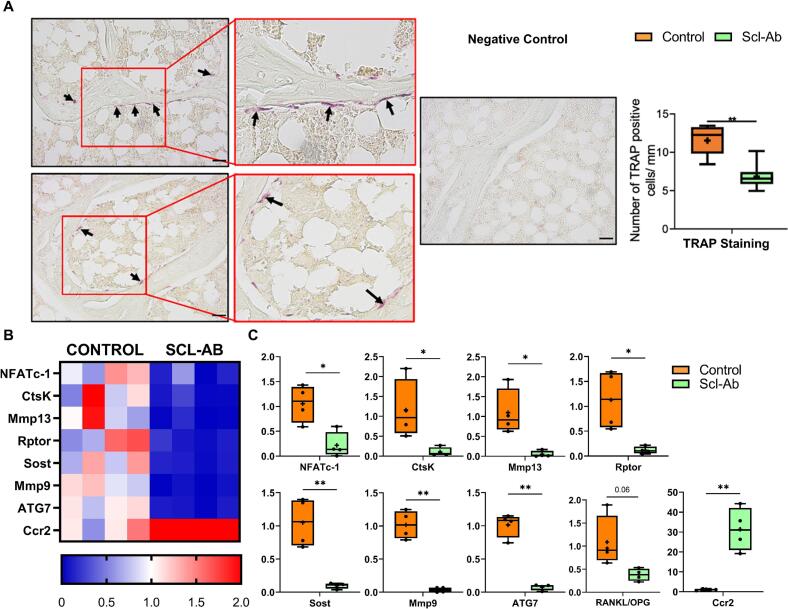
Osteoclastogenesis and bone resorption are reduced in OVX rats treated early with intermittent low-dose Scl-Ab. (A) TRAP+ staining is reduced in OVX animals that received subcutaneous Scl-Ab compared to untreated OVX animals (Control) at week 14. Representative images show TRAP staining (black arrows indicate TRAP+ osteoclasts) and fast green counterstaining of cortical bone in Control and Scl-Ab (magnified images in red boxes). ImageJ analysis was performed to quantify the number of TRAP+ cells per mm. (B) Heat map showing expression of genes involved in catabolic activity and matrix degradation in cortical bone from OVX rats at week 14, comparing untreated controls to those treated with 2 mg/kg/month Scl-Ab starting 3 weeks post-OVX. (C) Scl-Ab treatment significantly lowers expression of Sost, Nfatc1, Ctsk, Mmp9, Ccr2, and Rankl/Opg. *p < 0.05, **p < 0.01; *n* = 4/group. Scale = 20 μm. TRAP counts and histology are from distal femoral metaphyseal sections (trabecular compartment); gene expression data shown are from cortical bone of the proximal tibial diaphysis.

Early administration of intermittent low-dose Scl-Ab (2 mg/kg/month) significantly reduced osteoclast activity and gene expression associated with bone resorption. By week 14, TRAP+ staining was significantly lower in Scl-Ab-treated OVX animals than in untreated OVX controls (Fig. 2A).

Gene expression analysis confirmed a downregulation in key bone catabolism markers: NFATc-1 (6-fold), CtsK (12-fold), MMP13, Raptor, Sost (11-fold), MMP9 (25-fold), and ATG7 (10-fold) (Fig. 2B, C). A trend toward a lower RANKL/OPG ratio was observed in the Scl-Ab group (*p* = 0.06), suggesting a shift toward reduced osteoclastogenesis. Interestingly, CCR2 expression was upregulated (31-fold) in Scl-Ab-treated OVX animals compared to untreated controls (*p* < 0.05, Fig. 2B, C).

### 3.3 Osteocyte apoptosis and perilacunar remodelling are reduced in OVX rats treated early with intermittent low-dose Scl-Ab

**Fig. 3:**
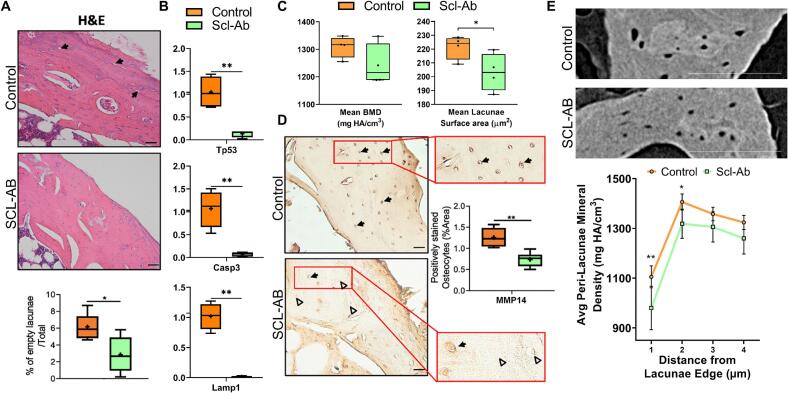
Osteocyte apoptosis and perilacunar remodelling are reduced in OVX rats treated early with intermittent low-dose Scl-Ab. (A) Scl-Ab administration results in a decrease in unoccupied lacunae. Representative images show Haematoxylin and Eosin (H&E) staining of cortical bone from OVX rats at week 14, comparing untreated controls to those treated with 2 mg/kg/month Scl-Ab starting 3 weeks post-OVX. ImageJ analysis was performed to quantify the percentage of empty lacunae (black arrows indicate empty lacunae). (B) Scl-Ab administration results in a decrease in gene expression associated with apoptosis (Tp53, Casp3, and Lamp1). (C) Mean BMD (mg HA/cm^**3**^), and Mean lacunae surface area (mm^**3**^) quantified using ultra-high-resolution nano-CT images. (D) Scl-Ab administration results in a decrease in MMP14+ osteocytes. Representative images show MMP14 staining in Control and Scl-Ab (magnified images, red boxes). Black arrows indicate MMP14+ osteocytes, and the white triangle indicates MMP14- osteocytes. ImageJ analysis was performed to assess the prevalence of positively stained osteocytes, normalized to total bone area. (E) Representative nanoCT images of metaphyseal trabecular bone from the proximal tibia from OVX rats at week 14, comparing untreated controls to those treated with 2 mg/kg/month Scl-Ab starting 3 weeks post-OVX, which were evaluated to determine the average peri-lacunae mineral density (mg HA/cm^**3**^), Scale bar = 20 μm (A and D) and 100 μm (E). *p < 0.05, **p < 0.01. H&E and MMP14 immunohistochemistry are from distal femoral cortical or metaphyseal sections as indicated; nano-CT lacunar/peri-lacunar measures and mean BMD are from cortical VOIs in the proximal tibia.

Scl-Ab treatment significantly reduced osteocyte apoptosis in OVX rats. Specifically, the percentage of empty lacunae was significantly lower in Scl-Ab-treated OVX animals compared to untreated OVX controls (*p* < 0.05, Fig. 3A). Apoptosis-related genes were downregulated in Scl-Ab-treated OVX animals: Tp53 (11-fold), Casp3 (11-fold), and Lamp1 (100-fold) (*p* < 0.01, Fig. 3B). Nano-CT revealed no significant difference in mean BMD between groups (*p* = 0.08; Fig. 3C). However, treated animals exhibited significantly smaller lacunar surface areas (Fig. 3C). The number of MMP14+ osteocytes, indicative of perilacunar remodelling, were significantly reduced in the Scl-Ab-treated group (*p* < 0.05, Fig. 3D). Mineral density significantly reduced near the lacunar edge in Scl-Ab-treated OVX animals compared to controls (*p* < 0.05; Fig. 3E). This reduction was localized, diminishing with distance from the lacunae (Fig. 3E).

### 3.4 Osteocyte gene expression is reduced in OVX rats early treated with intermittent low-dose Scl-Ab

**Fig. 4:**
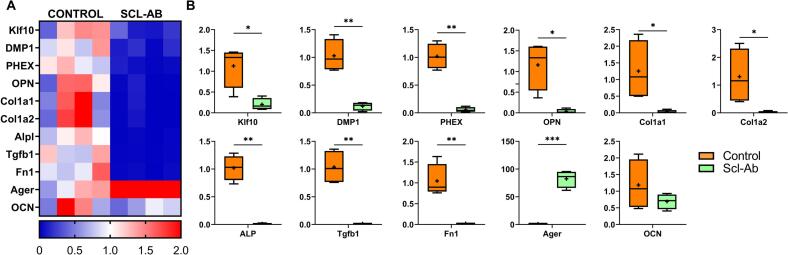
Scl-Ab administration decreases gene expression related to anabolism and bone matrix formation. (A) A heat map displaying anabolic and bone matrix formation gene expression in cortical bone from OVX rats at week 14, comparing untreated controls to those treated with 2 mg/kg/month Scl-Ab starting 3 weeks post-OVX. (B) Boxplots indicate a significant decrease in markers of anabolism and bone matrix formation (OPN, ALP, DMP1, PHEX) following Scl-Ab administration. n = 4 rats/group. *p < 0.05, **p < 0.01, ***p < 0.001. All gene expression data were obtained from cortical bone isolated from the proximal tibia (see Methods).

Scl-Ab-treated OVX animals exhibited significant downregulation of several osteocyte-enriched genes, including *Klf10* (6-fold), *DMP1*, and *PHEX* (10-fold each), which are critical for osteocyte-mediated regulation of mineral homeostasis and signalling within the bone matrix. Additionally, *OPN* (osteopontin), a matrix protein expressed by both osteoblasts and osteocytes, was significantly reduced (30-fold), suggesting a broader suppression of mineral-associated signalling within the osteocytic network. Importantly, expression of *AGER*, a receptor known to be upregulated in osteocytes under oxidative or inflammatory stress, was significantly increased (82-fold) in the Scl-Ab-treated group compared to untreated OVX controls (*p* < 0.05; Fig. 4A, B), pointing to potential activation of stress response pathways in osteocytes.

### 3.5 Osteoblast gene expression is reduced in OVX rats early treated with intermittent low-dose Scl-Ab

In parallel, we observed suppression of genes primarily associated with osteoblast function. This included downregulation of *ALP* (100-fold), a key enzyme involved in matrix mineralization, as well as *Col1α1* (43-fold) and *Col1α2* (65-fold), which encode the major structural components of type I collagen in the bone matrix. Additionally, expression of *TGFβ1* (50-fold) and *Fn1* (55-fold), both involved in osteoblast-mediated matrix signalling and remodelling, was significantly reduced (*p* < 0.01; Fig. 4A, B). No significant change was observed in *OCN* (osteocalcin) expression (*p* = 0.24), a late marker of osteoblast differentiation and bone formation.

### 3.6 Downregulation in mechanotransduction gene expression in OVX rats early treated with intermittent low-dose Scl-Ab

**Fig. 5:**
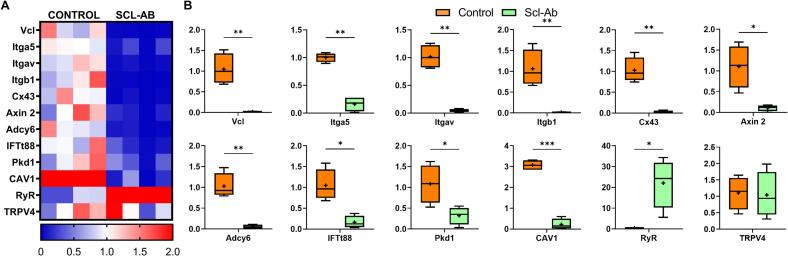
Scl-Ab Administration Downregulates Gene Expression Related to Mechanotransduction and Mechanosensation (A) A heat map displaying gene expression in cortical bone from OVX rats at week 14, comparing untreated controls to those treated with 2 mg/kg/month Scl-Ab starting 3 weeks post-OVX. (B) Boxplots reveal a significant decrease in markers of mechanotransduction and mechanosensation following Scl-Ab administration. n = 4 rats/group. *p < 0.05, **p < 0.01, ***p < 0.001. All gene expression data were obtained from cortical bone isolated from the proximal tibia (see Methods).

To explore the impact of Scl-Ab treatment on mechanotransduction pathways, we performed a heatmap analysis of related gene expression, which revealed downregulation in the Scl-Ab-treated OVX group: Vcl (55-fold), Integrin α5 (5-fold), Integrin αV (25-fold), Integrin β1 (50-fold), CX43 (33-fold), Axin2 (11-fold), Adcy6 (10-fold), IFT88 (6-fold), Pkd1 (4-fold), and Cav1 (16-fold) (p < 0.01, Fig. 5A, B). Conversely, RyR expression, a key regulator of intracellular calcium signalling, was significantly upregulated (22-fold) in Scl-Ab-treated OVX animals (*p* < 0.05, Fig. 5A, B). TRPV4 expression remained unchanged (*p* = 0.41).

## 4 Discussion

**Fig. 6:**
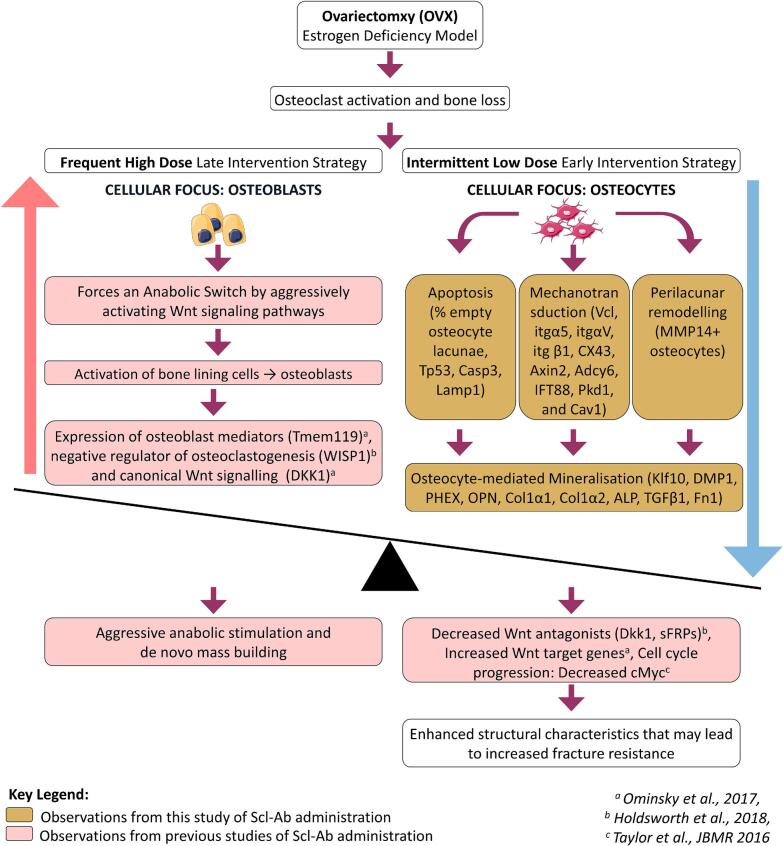
**Skeletal responses to sclerostin antibody** dosing strategy and timing of intervention may influence osteocyte responses and bone remodelling dynamics **during estrogen deficiency.** Early intermittent low-dose Scl-Ab (monthly) promotes trabecular surface bone formation and reduces osteoclastogenesis and osteocyte apoptosis, while reducing osteocyte-mediated mineral regulatory signalling and mechanotransduction gene expression. In contrast, published studies using sustained high-dose Scl-Ab (typically ≥25 mg/kg weekly) report robust anabolic responses characterized by increased osteogenic gene expression, inhibition of bone resorption and greater increases in bone mass.

The primary objective of this study was to assess the effectiveness of administering intermittent low-dose Sclerostin Antibody (Scl-Ab, 2 mg/kg/month) during the initial phase of rapid bone loss, prior to the onset of secondary mineralization changes (O'Sullivan et al., 2020). We report that early administration of intermittent low-dose Scl-Ab reduces osteoclastogenesis and osteocyte apoptosis while also limiting perilacunar remodelling, secondary mineralization, and changes in osteocyte mechanosensitivity associated with estrogen deficiency (see Fig. 6). Fluorochrome labelling revealed an increase in bone formation on trabecular surfaces following Scl-Ab therapy in OVX rats. This enhanced bone formation, combined with the suppression of osteoclastogenesis, resulted in an increase in bone volume. Collectively, these findings indicate that early, low-exposure sclerostin inhibition can mitigate bone loss while preserving osteocyte viability and modulating osteocyte-mediated mineralization.

Several limitations should be considered in this study. Firstly, the estrogen-deficient rat model may not fully replicate human osteoporosis, and individual reproductive histories were unavailable for these retired breeders. Nonetheless, the OVX rat is a widely established model of postmenopausal osteoporosis, because it recapitulates rapid bone loss following estrogen depletion and subsequent remodelling, and reproductive variability exists in the human population. Secondly, the effect of Scl-Ab injections on bone loss was less pronounced at the low dose (2 mg/kg) compared to the higher doses (25 mg/kg) reported to be effective in other OVX rat studies (Li et al., 2014). Additionally, the administration frequency in our study (once per month) differed from other studies, which typically administer Scl-Ab once or twice per week. Despite the lower cumulative exposure, a 2 mg/kg/month dose of Scl-Ab was sufficient to reduce osteoclast number, MMP14 expression in osteocytes, and the incidence of empty lacunae in OVX rats, demonstrating measurable efficacy even under conservative dosing conditions. Fourthly, mechanotransduction effects were inferred from gene expression and were not functionally validated, therefore interpretations regarding osteocyte signalling remain associative. The inclusion of mechanosensitive gene profiling nevertheless provides valuable insight into potential pathways involved in osteocytic responses to Scl-Ab. Also, the MAR was measured in vertebral trabecular bone, whereas other structural and molecular analyses were performed in the tibia and femur. Given the site-specific nature of bone adaptation to mechanical loading, pharmacological responses to Scl-Ab may differ between vertebral and long bones, and the vertebral MAR may not directly represent mineralization kinetics in the tibia or femur, where strain patterns and remodelling dynamics differ. Also, osteocyte morphology and TRAP^+^ osteoclasts were quantified in trabecular bone, whereas gene expression was analysed in cortical bone. Because remodelling activity and mechanical environments differ between trabecular and cortical compartments, cross-compartment comparisons should be interpreted with caution. Finally, direct experimental comparison between intermittent low-dose and sustained high-dose Scl-Ab regimens was beyond the scope of this study, therefore, dose-specific effects on osteocyte behaviour require confirmation in future work. Our approach was designed to probe osteocyte mechanobiological sensitivity under low-exposure conditions, rather than maximal pharmacological responses.

Our findings demonstrate that early administration of intermittent low-dose Scl-Ab in estrogen-deficient rats reduces osteoclastogenesis, which is consistent with its known dual effects on bone formation and resorption when the antibody is administered at higher doses (Ominsky et al., 2017a; Taylor et al., 2016). We observed reduced expression of NFATc-1, a key regulator of osteoclast function (Kim and Kim, 2014). Additionally, levels of CtsK (critical for bone resorption) and Raptor (a component of the mTOR pathway) were decreased (Dai et al., 2020; Dai et al., 2017). Together, these results indicate that even under conservative dosing conditions, early Scl-Ab exposure suppresses both osteoclast differentiation and resorptive activity. Although the magnitude of these effects was more modest than in high-dose studies, the observed increases in surface bone formation (as indicated by mineral apposition rate and bone formation rate), reductions in osteoclast activity and expression of resorption-associated markers, and improvements in trabecular bone mass areconsistent with reduced cumulative exposure.

Significant differences in trabecular bone mass and microarchitecture by week 14 align with established effects of Scl-Ab in mitigating bone loss in younger OVX rat models at higher and more frequent doses (25 and 50 mg/kg weekly), especially when administered at later stages (e.g., 8 weeks post-OVX) or in severe osteoporosis with unloading (Taylor et al., 2016; Zhang et al., 2016). Notably, in our study, Scl-Ab treatment began at three weeks post-OVX, near the end of the rapid bone loss phase, when 10–30% of bone mass had already been lost (O'Sullivan et al., 2020). While higher and more frequent regimens (25–50 mg/kg weekly) are known to elicit stronger pharmacodynamic effects in OVX rodents, these doses far exceed clinical exposure levels. The present study used a deliberately conservative monthly dose (2 mg/kg), which is lower than the approximate human-equivalent exposure (∼3 mg/kg/month, 210 mg romosozumab). This approach allowed us to assess osteocyte and bone-remodelling sensitivity under low-exposure conditions that may more closely resemble early therapeutic intervention rather than pharmacological saturation. The modest response observed is consistent with this conservative dosing strategy and provides insight into threshold-level efficacy and adaptive responses to intermittent sclerostin inhibition.

We report that early intermittent low-dose Scl-Ab treatment also reduces osteocyte apoptosis and the prevalence of empty lacunae, consistent with prior findings in glucocorticoid-induced osteoporosis models (Achiou et al., 2015). Moreover, Scl-Ab treatment led to reduced expression of apoptosis markers (Tp53, Casp3) and decreased lysosomal activity (Lamp1), suggesting a concurrent suppression of osteocyte death and its protective cellular responses. We also observed inhibition of MMP14-dependent perilacunar remodelling. These osteocyte-specific effects are likely linked to modulation of canonical Wnt/β-catenin signalling following sclerostin neutralization. Sclerostin is an inhibitor of Wnt signalling in osteocytes and its neutralization may enhance β-catenin activity within the osteocyte network (Li et al., 2005; Stegen et al., 2018; Tu et al., 2015; Fulzele et al., 2017; Allison et al., 2020), thereby promoting osteocyte survival and regulating perilacunar remodelling (Gortazar et al., 2013; Kramer et al., 2010; Jackson et al., 2021). Consistent with this mechanism, we report reduced expression of apoptosis-associated genes (Tp53 and Casp3), decreased lysosomal activity (Lamp1), and fewer empty lacunae in Scl-Ab–treated animals. Direct assessment of β-catenin activation and downstream Wnt targets would be required to confirm this proposed mechanism.

Micropetrosis commonly follows osteocyte apoptosis and is associated with mineral infilling of the osteocyte lacuna, which may be driven by a loss of osteocyte-mediated mineralization inhibitors. Thus, preservation of osteocyte viability may limit lacunar mineral infilling associated with micropetrosis. Perilacunar remodelling can be driven by osteocytes to degrade the local mineral matrix. In our study, intermittent low-dose Scl-Ab treatment led to a decrease in lacunar surface area and reduced local mineralization (< 2-μm) surrounding lacunae in OVX animals. These changes may be explained by osteocyte-driven processes, including micropetrosis or altered perilacunar remodelling. Further research is required to elucidate how Scl-Ab balances suppression of micropetrosis and perilacunar remodelling, and how this affects osteocyte regulation of their local microenvironment (Kogawa et al., 2018; Kogawa et al., 2013). The observed gene expression confirms that altered osteocyte viability occurs, which may occur independent to direct mineralization by osteoblasts.

We also observed a significant increase in mineral apposition rate, indicating enhanced osteoblast-driven surface bone formation in Scl-Ab-treated animals. However, anabolic gene expression in cortical bone was reduced at week 14. This apparent discrepancy likely reflects (1) temporal responses to treatment and (2) region-specific differences. Firstly, gene expression was evaluated at a relatively late time point (14 weeks post-OVX), whereas the anabolic response to sclerostin inhibition is temporally dynamic, with early increases in osteoblast proliferation and matrix synthesis followed by normalization of osteogenic gene expression during prolonged treatment (Boyce et al., 2018; Ominsky et al., 2015). Thus, we propose that the intermittent low-dose regimen elicited an early anabolic response at the bone surface, followed by adaptive normalization of gene expression by week 14. It is important to note that previous high-dose studies investigated gene expression at much earlier time points, ranging from hours to 1–2 weeks following Scl-Ab administration (Kim et al., 2017; Balani et al., 2021; Surowiec et al., 2020; Nioi et al., 2015), capturing acute transcriptional activation and early osteoprogenitor expansion. Secondly, we assessed surface bone formation in trabecular regions of the vertebra, which has a high osteoblast and progenitor density, whereas gene expression analyses were performed in cortical bone, which primarily contains osteocytes. Consequently, the transcriptional changes observed here may reflect osteocyte activity rather than osteoblast-driven bone formation occurring on trabecular surfaces. This pattern is consistent with transient osteoprogenitor proliferation described in prior high-dose Scl-Ab studies (Ominsky et al., 2010; Li et al., 2009)*,* although sustained upregulation of osteogenic gene expression at later stages has also been reported (Taylor et al., 2016; Li et al., 2014), including Col1α1. Direct temporal profiling and dose-comparison studies will be required to confirm this interpretation.

Importantly, while surface bone formation increased, osteocyte-mediated mineralization was reduced. To our knowledge, this is the first report showing downregulation of osteocyte-enriched genes (Klf10, DMP1, and PHEX) following early intermittent low-dose Scl-Ab treatment. Previous studies have reported that sclerostin antibody treatment alters bone matrix mineralization kinetics, including secondary mineral maturation within newly formed bone (Bala et al., 2010). In contrast, our results suggest that early low-dose Scl-Ab exposure may also influence osteocyte-mediated regulation of local mineral composition and perilacunar remodelling. These observations raise the possibility that osteocyte responses contribute to changes in bone mineral distribution following sclerostin inhibition.

Ovariectomy has previously been shown to increase osteocyte canalicular diameter (Allison et al., 2022), which may alter local fluid flow and the mechanical microenvironment of osteocytes (Allison et al., 2022; Ciani et al., 2014; Brennan et al., 2014; Tomkinson et al., 1998). Within this context, the observed reduction in Tp53, Casp3, and Lamp1 expression following intermittent low-dose Scl-Ab treatment supports a protective effect on osteocyte viability. Interestingly, a prior animal study reported increased p53 expression in osteocytes following Scl-Ab treatment (Taylor et al., 2016), suggesting that osteocyte responses to sclerostin inhibition may be context- or time-dependent. We also observed upregulation of AGER, a receptor associated with cellular signalling in response to changes in the extracellular matrix and mineral environment. This may reflect a compensatory response (Yang et al., 2021) to local alterations in matrix composition or mineral distribution induced by early intermittent low-dose Scl-Ab treatment. We also found downregulation of mechanotransduction-related genes (ItgαV, Itgβ1, and Cav1), which are essential for osteocyte mechanosensation and Wnt signalling. Short-term Scl-Ab treatment has also been associated with reduced osteocyte mechanosensitivity, possibly due to the presence of immature osteocytes following new bone formation (Morrell et al., 2021). However, longer-term studies show a return to normal osteocyte signalling, which may explain the eventual reduction in bone formation (Morrell et al., 2021). Early sclerostin inhibition may therefore transiently alter osteocyte mechanosensitivity during periods of active bone remodelling. We propose that Scl-Ab may initially reduce osteocyte sensitivity to mechanical loading as an adaptive response to changes in the local mechanical environment following bone loss remodelling. By dampening mechanotransduction, Scl-Ab may help limit osteocyte apoptosis and pathological mineralization (Verbruggen et al., 2015). However, these findings are correlative, we assessed gene expression changes in cortical bone but did not perform functional loading experiments. Future studies combining controlled mechanical loading with real-time mechanosignalling measurements will be required to directly test this hypothesis.

In summary, early intermittent low-dose Scl-Ab treatment enhances surface bone formation, reduces bone resorption, and preserves osteocyte viability, while modulating osteocyte-mediated mineralization and perilacunar remodelling during estrogen deficiency. These findings suggest that the osteocyte network may represent an early and highly sensitive target of sclerostin inhibition. Whether intermittent low-dose Scl-Ab regimens can sustain bone formation efficiency over longer durations and avoid the anabolic attenuation reported with higher-dose treatment remains unclear and requires direct longitudinal investigation.

## CRediT authorship contribution statement

**Syeda Masooma Naqvi:** Writing – review & editing, Writing – original draft, Visualization, Validation, Methodology, Investigation, Formal analysis, Data curation, Conceptualization. **Wahaaj Ali:** Writing – review & editing, Visualization, Methodology, Investigation, Formal analysis, Conceptualization. **Hollie Allison:** Visualization, Validation, Methodology, Investigation, Formal analysis, Data curation. **Laura M. O'Sullivan:** Visualization, Validation, Methodology, Investigation, Formal analysis, Data curation. **Gill Holdsworth:** Writing – review & editing. **Juan Alberto Panadero-Perez:** Writing – review & editing, Validation, Methodology, Investigation. **Jessica Schiavi-Tritz:** Writing – review & editing, Validation, Methodology, Investigation. **Laoise M. McNamara:** Writing – review & editing, Writing – original draft, Supervision, Resources, Project administration, Methodology, Investigation, Funding acquisition, Conceptualization.

## Declaration of competing interest

The authors declare the following financial interests and personal relationships that may be considered potential competing interests: this study was supported by provision of sclerostin antibody from UCB Ltd. Gill Holdsworth reports a relationship with UCB Ltd. that includes employment.

## Data Availability

Data will be made available on request.

## Appendix A Supplementary data

Supplementary Figure 1 (A) Sample preparation for nano-CT imaging. A ∼0.5 mm slice was extracted from the whole bone using a diamond blade. (B) A rendered VOI from a representative sample, with segmentation and labelling of individual trabecular structures. (C) Peri-lacunar mineral density assessment. Lacunae ROIs were dilated by 2 pixels, with the inner lacunar volume removed to isolate the peri-lacunar region for each lacuna. Lacunae are segmented in red, and peri-lacunar regions are segmented in blue. (D) Peri-lacunar mineral density variation. A similar strategy was used to analyse peri-lacunar mineral variation. New ROIs were defined by dilating the original ROI by 1 pixel at 1.1-micron spacing. Lacunae are segmented in red, with peri-lacunar regions visualized in sequentially expanded layers.

## References

[bb0005] Achiou Z. (2015). Sclerostin antibody and interval treadmill training effects in a rodent model of glucocorticoid-induced osteopenia. Bone.

[bb0010] Allison H., Holdsworth G., McNamara L.M. (2020). Scl-ab reverts pro-osteoclastogenic signalling and resorption in estrogen deficient osteocytes. BMC Molecular and Cell Biology.

[bb0015] Allison H., McNamara L.M. (2019). Inhibition of osteoclastogenesis by mechanically stimulated osteoblasts is attenuated during estrogen deficiency. Am. J. Phys. Cell Phys..

[bb0020] Allison H., O’Sullivan L.M., McNamara L.M. (2022). Temporal changes in cortical microporosity during estrogen deficiency associated with perilacunar resorption and osteocyte apoptosis: a pilot study. Bone Reports.

[bb0025] Bala Y. (2010). Time sequence of secondary mineralization and microhardness in cortical and cancellous bone from ewes. Bone.

[bb0030] Balani D.H. (2021). Sclerostin antibody administration increases the numbers of Sox9creER+ skeletal precursors and their progeny. J. Bone Miner. Res..

[bb0035] Bonewald L.F. (2011). The amazing osteocyte. J. Bone Miner. Res..

[bb0040] Boyce R.W. (2018). Decreased osteoprogenitor proliferation precedes attenuation of cancellous bone formation in ovariectomized rats treated with sclerostin antibody. Bone Rep.

[bb0045] Brennan M.A. (2014). Estrogen withdrawal from osteoblasts and osteocytes causes increased mineralization and apoptosis. Horm. Metab. Res..

[bb0050] Ciani C. (2014). Ovariectomy enhances mechanical load-induced solute transport around osteocytes in rat cancellous bone. Bone.

[bb0055] Cosman F. (2016). Romosozumab treatment in postmenopausal women with osteoporosis. N. Engl. J. Med..

[bb0060] Costa S. (2021). Sclerostin antibody increases trabecular bone and bone mechanical properties by increasing osteoblast activity damaged by whole-body irradiation in mice. Bone.

[bb0065] Dai Q. (2017). Inactivation of regulatory-associated protein of mTOR (raptor)/mammalian target of rapamycin complex 1 (mTORC1) signaling in osteoclasts increases bone mass by inhibiting osteoclast differentiation in mice*. J. Biol. Chem..

[bb0070] Dai R. (2020). Cathepsin k: the action in and beyond bone. Front. Cell Dev. Biol..

[bb0075] Deepak V., Kayastha P., McNamara L.M. (2017). Estrogen deficiency attenuates fluid flow-induced [Ca2+]i oscillations and mechanoresponsiveness of MLO-Y4 osteocytes. FASEB J..

[bb0080] Dole N.S. (2017). Osteocyte-intrinsic TGF-β signaling regulates bone quality through Perilacunar/Canalicular remodeling. Cell Rep..

[bb0085] Emerton K.B. (2010). Osteocyte apoptosis and control of bone resorption following ovariectomy in mice. Bone.

[bb0090] Fulzele K. (2017). Osteocyte-secreted Wnt signaling inhibitor Sclerostin contributes to beige Adipogenesis in peripheral fat depots. J. Bone Miner. Res..

[bb0095] Geoghegan I.P., Hoey D.A., McNamara L.M. (2019). Estrogen deficiency impairs integrin αvβ3-mediated mechanosensation by osteocytes and alters osteoclastogenic paracrine signalling. Sci. Rep..

[bb0100] Gortazar A.R. (2013). Crosstalk between caveolin-1/extracellular signal-regulated kinase (ERK) and β-catenin survival pathways in osteocyte mechanotransduction. J. Biol. Chem..

[bb0105] Gregson C.L. (2022). UK clinical guideline for the prevention and treatment of osteoporosis. Arch. Osteoporos..

[bb0110] Holdsworth G. (2018). Dampening of the bone formation response following repeat dosing with sclerostin antibody in mice is associated with up-regulation of Wnt antagonists. Bone.

[bb0115] Jackson E. (2021). Osteocyte Wnt/β-catenin pathway activation upon mechanical loading is altered in ovariectomized mice. Bone Rep.

[bb0120] Kedlaya R. (2013). Sclerostin inhibition reverses skeletal fragility in an Lrp5-deficient mouse model of OPPG syndrome. Sci. Transl. Med..

[bb0125] Kim J.H., Kim N. (2014). Regulation of NFATc1 in osteoclast differentiation. J Bone Metab.

[bb0130] Kim S.W. (2017). Sclerostin antibody administration converts bone lining cells into active osteoblasts. J. Bone Miner. Res..

[bb0135] Kogawa M. (2013). Sclerostin regulates release of bone mineral by osteocytes by induction of carbonic anhydrase 2. J. Bone Miner. Res..

[bb0140] Kogawa M. (2018). Recombinant sclerostin antagonizes effects of ex vivo mechanical loading in trabecular bone and increases osteocyte lacunar size. Am. J. Phys. Cell Phys..

[bb0145] Kramer I. (2010). Osteocyte Wnt/beta-catenin signaling is required for normal bone homeostasis. Mol. Cell. Biol..

[bb0150] Li X. (2005). Sclerostin binds to LRP5/6 and antagonizes canonical Wnt signaling. J. Biol. Chem..

[bb0155] Li X. (2009). Sclerostin antibody treatment increases bone formation, bone mass, and bone strength in a rat model of postmenopausal osteoporosis. J. Bone Miner. Res..

[bb0160] Li X. (2014). Progressive increases in bone mass and bone strength in an ovariectomized rat model of osteoporosis after 26 weeks of treatment with a sclerostin antibody. Endocrinology.

[bb0165] McClung M.R. (2014). Romosozumab in postmenopausal women with low bone mineral density. N. Engl. J. Med..

[bb0170] McClung M.R. (2018). Effects of 24 months of treatment with Romosozumab followed by 12 months of Denosumab or placebo in postmenopausal women with low bone mineral density: a randomized, double-blind, phase 2, parallel group study. J. Bone Miner. Res..

[bb0175] Morrell A.E. (2021). Osteocyte mechanosensing following short-term and long-term treatment with sclerostin antibody. Bone.

[bb0180] Naqvi S.M. (2024). Altered extracellular matrix and mechanotransduction gene expression in rat bone tissue following long-term estrogen deficiency. JBMR Plus.

[bb0185] Nioi P. (2015). Transcriptional profiling of laser capture microdissected subpopulations of the osteoblast lineage provides insight into the early response to Sclerostin antibody in rats. J. Bone Miner. Res..

[bb0190] Notsu M. (2017). Advanced glycation end product 3 (AGE3) increases apoptosis and the expression of Sclerostin by stimulating TGF-β expression and secretion in osteocyte-like MLO-Y4-A2 cells. Calcif. Tissue Int..

[bb0195] Ominsky M.S. (2010). Two doses of sclerostin antibody in cynomolgus monkeys increases bone formation, bone mineral density, and bone strength. J. Bone Miner. Res..

[bb0200] Ominsky M.S. (2015). Differential temporal effects of sclerostin antibody and parathyroid hormone on cancellous and cortical bone and quantitative differences in effects on the osteoblast lineage in young intact rats. Bone.

[bb0205] Ominsky M.S. (2017). Effects of sclerostin antibodies in animal models of osteoporosis. Bone.

[bb0210] Ominsky M.S. (2017). Romosozumab improves bone mass and strength while maintaining bone quality in Ovariectomized Cynomolgus monkeys. J. Bone Miner. Res..

[bb0215] O’Sullivan L.M. (2020). Secondary alterations in bone mineralisation and trabecular thickening occur after long-term estrogen deficiency in ovariectomised rat tibiae, which do not coincide with initial rapid bone loss. Osteoporos. Int..

[bb0220] Padhi D. (2011). Single-dose, placebo-controlled, randomized study of AMG 785, a sclerostin monoclonal antibody. J. Bone Miner. Res..

[bb0225] Padhi D. (2014). Multiple doses of sclerostin antibody romosozumab in healthy men and postmenopausal women with low bone mass: a randomized, double-blind, placebo-controlled study. J. Clin. Pharmacol..

[bb0230] Parle E. (2020). Bone mineral is more heterogeneously distributed in the femoral heads of osteoporotic and diabetic patients: a pilot study. JBMR Plus.

[bb0235] Ross R.D. (2014). Bone matrix quality after sclerostin antibody treatment. J. Bone Miner. Res..

[bb0240] Saag K.G. (2017). Romosozumab or alendronate for fracture prevention in women with osteoporosis. N. Engl. J. Med..

[bb0245] Simfia I., Schiavi J., McNamara L.M. (2020). Alterations in osteocyte mediated osteoclastogenesis during estrogen deficiency and under ROCK-II inhibition: an in vitro study using a novel postmenopausal multicellular niche model. Exp. Cell Res..

[bb0250] Sowers M.R. (2010). Amount of bone loss in relation to time around the final menstrual period and follicle-stimulating hormone staging of the transmenopause. J. Clin. Endocrinol. Metab..

[bb0255] Stegen S. (2018). Osteocytic oxygen sensing controls bone mass through epigenetic regulation of sclerostin. Nat. Commun..

[bb0260] Stolina M. (2014). Temporal changes in systemic and local expression of bone turnover markers during six months of sclerostin antibody administration to ovariectomized rats. Bone.

[bb0265] Surowiec R.K. (2020). Gene expression profile and acute gene expression response to Sclerostin inhibition in osteogenesis imperfecta bone. JBMR Plus.

[bb0270] Taylor S. (2016). Time-dependent cellular and transcriptional changes in the osteoblast lineage associated with sclerostin antibody treatment in ovariectomized rats. Bone.

[bb0275] Tomkinson A. (1998). The role of estrogen in the control of rat osteocyte apoptosis. J. Bone Miner. Res..

[bb0280] Tu X. (2015). Osteocytes mediate the anabolic actions of canonical Wnt/beta-catenin signaling in bone. Proc. Natl. Acad. Sci. USA.

[bb0285] Turk J.R. (2020). Nonclinical cardiovascular safety evaluation of romosozumab, an inhibitor of sclerostin for the treatment of osteoporosis in postmenopausal women at high risk of fracture. Regul. Toxicol. Pharmacol..

[bb0290] Verbruggen S.W. (2015). Altered mechanical environment of bone cells in an animal model of short- and long-term osteoporosis. Biophys. J..

[bb0295] Virdi A.S. (2015). Sclerostin antibody treatment improves implant fixation in a model of severe osteoporosis. J. Bone Joint Surg. Am..

[bb0300] Wu J. (2018). The effects of sclerostin antibody plus parathyroid hormone (1-34) on bone formation in ovariectomized rats. Z. Gerontol. Geriatr..

[bb0305] Yang X. (2021). Effects of advanced glycation end products on osteocytes mechanosensitivity. Biochem. Biophys. Res. Commun..

[bb0310] Zhang D.Y. (2016). Sclerostin antibody prevented progressive bone loss in combined ovariectomized and concurrent functional disuse. Bone.

