# Supplementary material for: Early intermittent low-dose sclerostin antibody treatment promotes surface bone formation and reduces bone loss, but also decreases osteocyte apoptosis and mechanotransduction in ovariectomized rats: A pilot study
Source: Bone Rep. 2026 Apr 7;29:101915. doi: 10.1016/j.bonr.2026.101915 (PMC13101557; doi:10.1016/j.bonr.2026.101915)
Supplement: Supplementary Figure 1 — (A) Sample preparation for nano-CT imaging. A ∼0.5 mm slice was extracted from the whole bone using a diamond blade. (B) A rendered VOI from a representative sample, with segmentation and labelling of individual trabecular structures. (C) Peri-lacunar mineral density assessment. Lacunae ROIs were dilated by 2 pixels, with the inner lacunar volume removed to isolate the peri-lacunar region for each lacuna. Lacunae are segmented in red, and peri-lacunar regions are segmented in blue. (D) Peri-lacunar mineral density variation. A similar strategy was used to analyse peri-lacunar mineral variation. New ROIs were defined by dilating the original ROI by 1 pixel at 1.1-micron spacing. Lacunae are segmented in red, with peri-lacunar regions visualized in sequentially expanded layers. [file mmc1.docx]

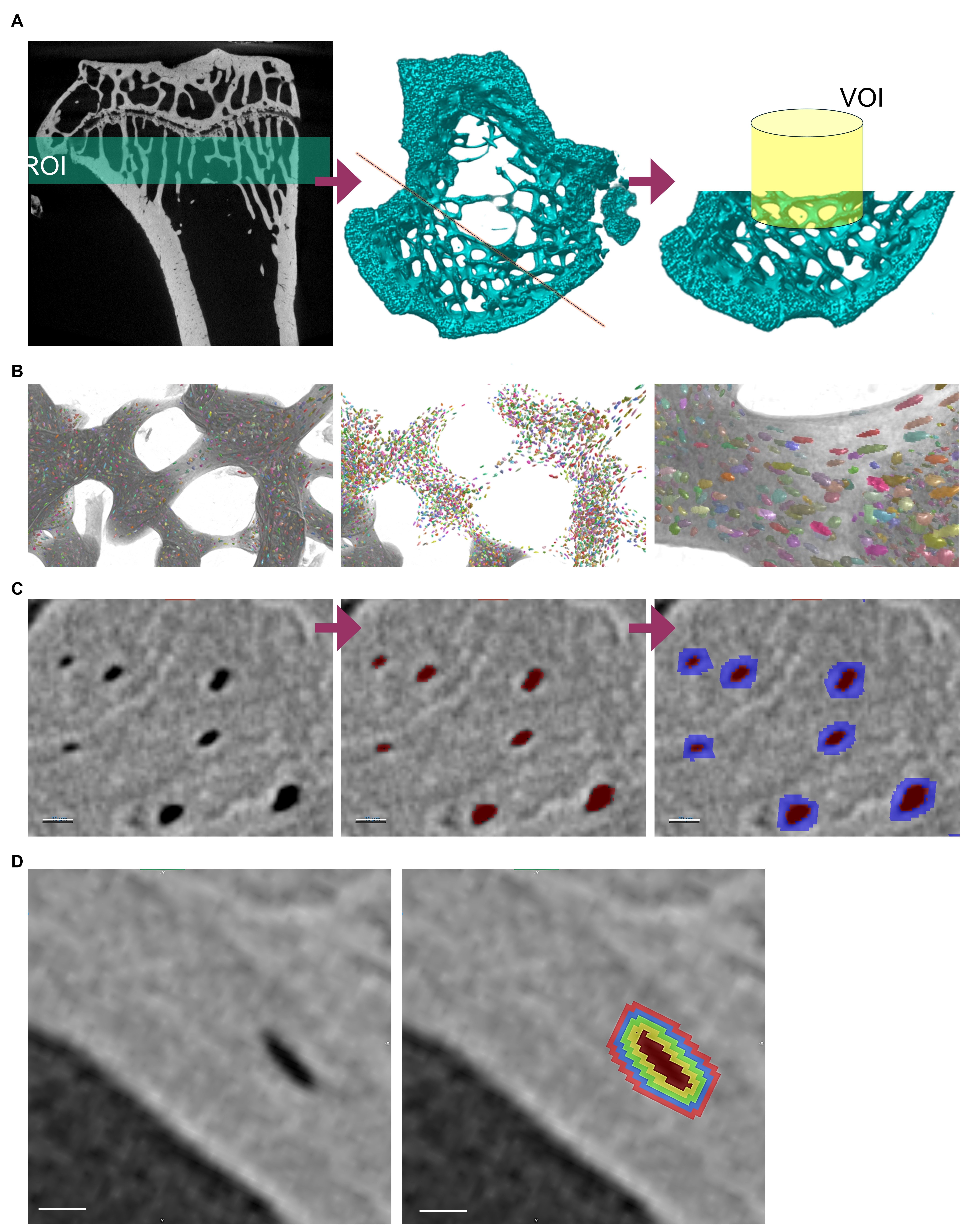
**Supplementary Figure 1:** *(A) Sample preparation for nano-CT imaging. A ~0.5 mm slice was extracted from the whole bone using a diamond blade. (B) A rendered VOI from a representative sample, with segmentation and labelling of individual trabecular structures. (C) Peri-lacunar mineral density assessment. Lacunae ROIs were dilated by 2 pixels, with the inner lacunar volume removed to isolate the peri-lacunar region for each lacuna. Lacunae are segmented in red, and peri-lacunar regions are segmented in blue. (D) Peri-lacunar mineral density variation. A similar strategy was used to analyse peri-lacunar mineral variation. New ROIs were defined by dilating the original ROI by 1 pixel at 1.1-micron spacing. Lacunae are segmented in red, with peri-lacunar regions visualized in sequentially expanded layers.*
